# Neck control after definitive radiochemotherapy without planned neck dissection in node-positive head and neck cancers

**DOI:** 10.1186/1471-2407-12-59

**Published:** 2012-02-07

**Authors:** Na Young Jang, Keun-Wook Lee, Soon-Hyun Ahn, Jae-Sung Kim, In Ah Kim

**Affiliations:** 1 Department of Radiation Oncology, Seoul National University Bundang Hospital, 300 Gumi-ro, Bundang-gu, Seongnam-si, Gyeonggi-do 463-707, Korea; 2 Internal Medicine, Seoul National University Bundang Hospital, 300 Gumi-ro, Bundang-gu, Seongnam-si, Gyeonggi-do 463-707, Korea; 3 Otorhinolaryngology, Seoul National University Bundang Hospital, 300 Gumi-ro, Bundang-gu, Seongnam-si, Gyeonggi-do 463-707, Korea

## Abstract

**Background:**

The purpose of this study was to evaluate neck control outcomes after definitive radiochemotherapy without planned neck dissection in node-positive head and neck cancer.

**Methods:**

We retrospectively reviewed medical records of fifty patients with node-positive head and neck cancer who received definitive radiochemotherapy. Twelve patients subsequently underwent neck dissection for suspicious recurrent or persistent disease. A median dose of 70 Gy (range 60-70.6) was delivered to involved nodes. Response evaluation was performed at a median of 5 weeks after completion of radiotherapy.

**Results:**

Neck failure was observed in 11 patients and the 3-year regional control (RC) rate was 77.1%. Neck dissection was performed in 10 of the 11 patients; seven of these cases were successfully salvaged, and the ultimate rate of neck control was 92%. The remaining two patients who received neck dissection had negative pathologic results. On univariate analysis, initial nodal size > 2 cm, a less-than-complete response at the primary site, post-radiotherapy nodal size > 1.5 cm, and post-radiotherapy nodal necrosis were associated with RC. On multivariate analysis, less-than-complete primary site response and post-radiotherapy nodal necrosis were identified as independent prognostic factors for RC.

**Conclusions:**

The neck failure rate after definitive radiochemotherapy without planned neck dissection was 22%. Two-thirds of these were successfully salvaged with neck dissection and the ultimate neck control rate was 92%. Our results suggest that planned neck dissection might not be necessary in patients with complete response of primary site, no evidence of residual lesion > 1.5 cm, or no necrotic lymph nodes at the 1-2 months follow-up evaluation after radiotherapy.

## Background

Radiotherapy combined with systemic therapy results in increased locoregional control with organ preservation in locally advanced head and neck cancers [1-4]. However, there has been controversy over neck treatment after definitive radiochemotherapy; specifically, whether to perform a subsequent "planned" neck dissection (regardless of nodal response) or only "salvage" neck dissection for persistent or recurrent disease [5]. The rationale for routine planned neck dissection is that pathological positivity rates are high (30%-40%) in post-radiochemotherapy neck dissections [6,7] and the majority of patients who experience a neck recurrence are unlikely to be successfully salvaged [8]. The rationale for observation and salvage neck dissection is the concern of overtreatment because additional surgery for all patients is unlikely to increase regional control rate [5].

There are some studies reporting the results of omitting planned neck dissection in patients who obtain complete response (defined ambiguously as "not clinically detectable"). Their regional failure rate was generally below 10%, similar to regional control rates in planned neck dissection series [5]. The problem here is defining the criteria for "detectable disease".

At Seoul National University Bundang Hospital, the standard treatment policy is "salvage" neck dissection for persistent or recurrent disease. Because there were no definite criteria of complete nodal response, we included all patients treated with definitive radiochemotherapy regardless of nodal response. This study describes our experience treating patients with node-positive head and neck cancer with definitive radiochemotherapy without planned neck dissection. We investigated the patterns of failure, neck control rate, and prognostic factors for regional control to evaluate the clinical outcome of our treatment policy.

## Methods

We retrospectively reviewed the medical records of 50 patients with node positive head and neck cancer who received definitive radiochemotherapy without immediate planned neck dissection between June 2003 and August 2010 at Seoul National University Bundang Hospital.

Patients and tumor characteristics are listed in Table 1. The study population was mostly male (82%), with a median age of 57 years. Clinical tumor and nodal staging (according to the 7th edition of American Joint Committee on Cancer staging system) was evaluated by physical and endoscopic examination, computed tomography (CT) and/or magnetic resonance imaging (MRI) and^18^F-fludeoxyglucose (FDG) positron emission tomography (PET). Fine needle aspiration (FNA) for suspicious lymph nodes was performed in 23 patients. Lymph node with short diameter ≥1 cm (long diameter ≥0.8 cm in case of retropharyngeal node) was considered pathologic node but it was not a strict criteria. Lymph node with necrosis, abnormal FDG uptake, or positive for malignancy by FNA was considered pathologic node regardless of its size. For PET interpretation, there was no strict cut off maximum standardized values (mSUV). An experienced nuclear medicine physician interpreted the PET/CT images by visual inspection. Foci of increased FDG uptake were evaluated by comparison with background and blood pool activity.

**Table 1:** Patient and tumor characteristics

Variable	**No**.	%
Age (years)	Median 57 (range 38-74)	
Gender		
Male	41	82
Female	9	18
Primary site		
Nasopharynx	25	50
Oropharynx	12	24
Hypopharynx	9	18
Larynx	4	8
T classification		
1	14	28
2	19	38
3	8	16
4	9	18
N classification		
1	12	24
2	30	60
3	8	16
Stage		
2	7	14
3	14	28
4	29	58
Histology		
Squamous cell carcinoma	25	50
Nonkeratinizing undifferentiated carcinoma	22	44
Nonkeratinizing differentiated carcinoma	1	2
Keratinizing squamous cell carcinoma	2	4
Differentiation		
Well differentiated	3	6
Moderate differentiated	8	16
Poorly differentiated	6	12
Undifferentiated	22	44
Not available	11	22
Initial maximal lymph node size (cm)	Median 2 (range 1-7)	

Twenty-nine patients received conventional (n = 4) or 3-dimensional conformal (n = 25) radiotherapy (3D-CRT) with a median dose of 70 Gy (range 60-70.6 Gy) to the primary tumor and involved nodes and 50 Gy to the elective nodal area. Twenty-one patients received intensity-modulated radiotherapy (IMRT) with a simultaneous integrated boost (SIB) technique. The dose prescription of IMRT was as follows: 67.5 Gy at 2.25 Gy/fraction to gross tumor (primary tumor and involved nodes), 54 Gy at 1.8 Gy/fraction to subclinical disease, and 49.5 Gy at 1.65 Gy/fraction to elective neck. The chemotherapy administration sequences varied: neoadjuvant/concurrent in 19 patients, concurrent/adjuvant in 13 patients, concurrent only in 12 patients, neoadjuvant/concurrent/adjuvant in four patients, and neoadjuvant only in two patients. Various cisplatin-based regimens were administered. For neoadjuvant chemotherapy, 1-3 cycles of 5-flurouracil 1,000-1,200 mg/m^2 ^on day 1-4 plus cisplatin 60-80 mg/m^2 ^on day 1 (FP, n = 8), 1-4 cycles of docetaxel 75 mg/m^2 ^plus cisplatin 75 mg/m^2 ^on day 1 (DP, n = 7), 1-3 cycles of docetaxel 75 mg/m^2 ^on day 1, 5-FU 1,000 mg/m^2 ^on day 1-3, plus cisplatin 75 mg/m^2 ^on day 2-3 (DFP, n = 5), or 3 cycles of DP plus cetuximab (400 mg/m^2 ^on day 1 as first dose and then 250 mg/m^2 ^weekly) (n = 5) were used. For concurrent chemotherapy, 2-3 cycles of 3-weekly cisplatin 100 mg/m^2 ^on day 1 (n = 19), weekly cisplatin 30 mg/m^2 ^(n = 17), weekly cetuximab 250 mg/m^2 ^(400 mg/m^2 ^as first dose) (n = 6), weekly cisplatin 30 mg/m^2 ^plus cetuximab 250 mg/m^2 ^(n = 4), or 2 cycles of FP (n = 2) were used. Two to four cycles of adjuvant FP were administered to 17 patients until March 2008. Immediate planned neck dissection was not performed after radiochemotherapy. Twelve patients who had suspicious persistent or recurrent disease during follow-up underwent subsequent neck dissection.

First response evaluation was performed by physical and endoscopic examination, CT, or MRI at a median of 5 weeks after completion of radiotherapy (range 1-14, interquartile range 4-7). PET was performed at the first follow-up in 31 patients. Follow-up physical and endoscopic examinations were performed every 1-2 months for the first year, every 3 months for years 2-3, every 6 months for years 4-5, and every 6-12 months thereafter. Imaging studies were performed every 3-4 months for the first 2 years and then every 6-12 months. The primary tumor responses were evaluated according to the Response Evaluation Criteria in Solid Tumors criteria (RECIST) [9]. Because there were no definite criteria of complete nodal response among studies regarding observation and salvage neck dissection, we included all patients treated with definitive radiochemotherapy regardless of nodal response. If involved nodes were all disappeared or reduced to < 0.5 cm (difficult to measure the correct size), we observed the patient with confidence. If there was an increase of ≥20% diameter in any node or appearance of new lesion, we considered it progression and performed salvage neck dissection. However, in most other cases, we deferred the decision and observed closely in next 1-3 months. If the lymph node size further decreased or was stable with short diameter < 1 cm without necrosis or abnormal FDG uptake at second examination, the patient was observed by above protocol. One patient who achieved partial response in primary tumor and lymph node underwent early neck dissection (at 10 weeks postoperatively). The patient's lymph node was 6.5 cm initially, and 3.8 cm at post-radiotherapy 4 weeks. At 10 weeks post-radiotherapy, residual node was still 3.8 cm and salvage neck dissection was performed for persistent disease. During follow-up, increase of ≥20% diameter compared with prior smallest size in any single node or appearance of new lesion was considered recurrent nodal disease and salvage treatment was performed.

Overall survival (OS), recurrence-free survival (RFS), local control (LC), regional control (RC), and distant control (DC) rates were calculated using the Kaplan-Meier method. Data for patients who were alive or dead without each type of recurrence were used as censored data in calculating LC/RC/DC rates. Prognostic factors such as age, gender, primary site, T/N classification, initial lymph node size, radiotherapy modality, chemotherapy administration sequence, primary tumor response, post-radiotherapy lymph node size/necrosis, and PET reading of positive residual nodal disease were evaluated using log-rank statistics. Among the factors, those with *p *< 0.2 were selected and included in the multivariate regression analysis using the Cox proportional hazards regression model. The statistical analysis was performed with PASW version 17.0 (SPSS Inc., Chicago, IL).

## Results

**Figure 1:**
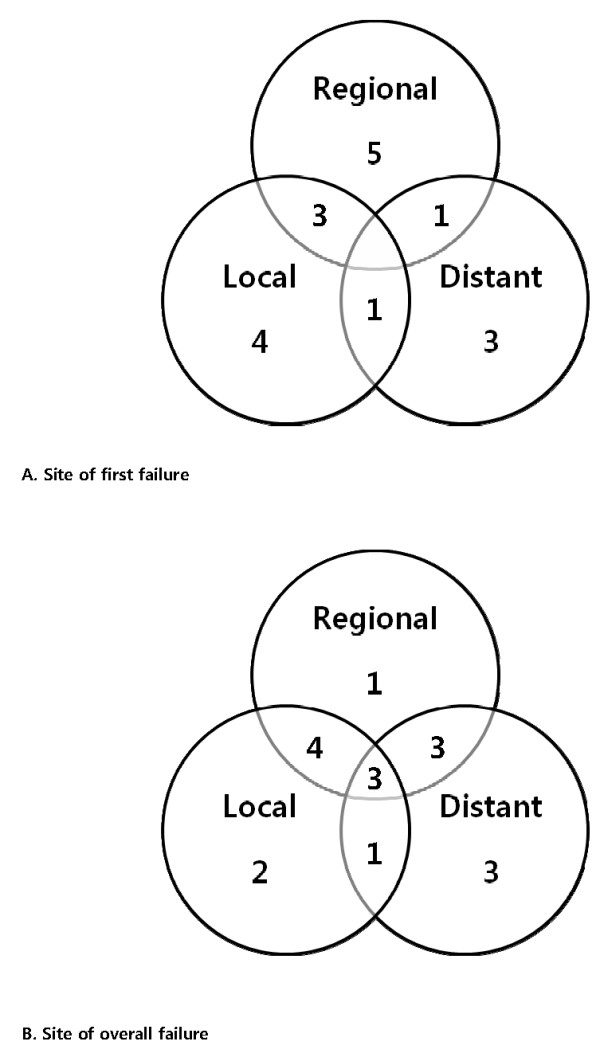
**Patterns of (A) first failure and (B) overall failure**. Of the five patients who experienced regional recurrence as an isolated first recurrence, two experienced subsequent distant metastasis and two experienced subsequent local and distant failure. Of the four patients who experienced local recurrence as an isolated first recurrence, one patient experienced subsequent regional failure and one experienced subsequent regional and distant failure.

The median follow-up duration after radiotherapy was 36.7 months (range 14.0-90.5). A total of 17 patients experienced recurrences and two patients died. The patterns of first failure and overall failure at the time of the last follow-up are shown in Figure 1. Of the five patients who experienced regional recurrence as an isolated first recurrence, two experienced subsequent distant metastasis and two experienced subsequent local and distant failure. Of the four patients who experienced local recurrence as an isolated first recurrence, one patient experienced subsequent regional failure and one experienced subsequent regional and distant failure. Overall local, regional, and distant failure developed in 10, 11, and 10 patients, respectively. The 3-year OS, RFS, LC, RC, and DC rates were 95.7%, 62.7%, 77.2%, 77.1%, and 78.2%, respectively.

Regional recurrence developed in 11 patients (crude rate 22%) at a median of 4.9 months after radiotherapy (range 2.3-23.6, interquartile range 2.6-7.0). Ipsilateral level II was the most common site of regional failure. Neck dissection for suspicious persistent or recurrent disease was performed in 12 patients at 2.3-24.5 months (median 6 months) after radiotherapy. Of these, two patients had no metastatic lymph nodes and 10 had metastatic lymph nodes in the neck. Of these 10 patients, seven were successfully salvaged, and the ultimate neck control rate was 92%. With the exception of two patients who developed distant metastasis, the other five of these seven patients were alive without disease at the last follow-up. Two patients with isolated local failure and one patient with isolated lung metastasis were also successfully salvaged with surgery and chemotherapy. Thus, 8 of 17 patients with recurrence were alive without disease at the last follow-up (median 24.6 months, range 9.7-88.1 months after recurrence).

We evaluated candidate parameters such as age, gender, primary site, T/N classification, initial lymph node size, radiotherapy modality, chemotherapy administration sequence, primary tumor response, post-radiotherapy lymph node size/necrosis, and PET reading of positive residual nodal disease to identify prognostic factors for regional control, and the results are listed in Table 2. On univariate analysis, initial nodal size > 2 cm (3-year RC 88.0% vs. 63.6%, p = 0.022), post-radiotherapy primary tumor response less than complete response (88.1% vs. 41.7%, p < 0.001), post-radiotherapy nodal size > 1.5 cm (83.5% vs. 20.0%, p < 0.001), and post-radiotherapy nodal necrosis (87.8% vs. 33.3%, p = 0.002) were associated with poor regional control. Administration of chemotherapy was heterogeneous and we divided patients into 3 groups; neoadjuvant plus concurrent, concurrent plus adjuvant, and concurrent only; corresponding 3-year RC rates were 71.4%, 94.1%, and 64.3%, respectively (p = 0.123). On multivariate analysis, a less-than-complete primary site response (hazard ratio 8.926, 95% confidence interval 2.38-33.47, p = 0.001) and post-radiotherapy nodal necrosis (hazard ratio 7.413, 95% confidence interval 2.03-27.14, p = 0.002) were identified as independent prognostic factors for regional control.

**Table 2:** Univariate analysis to identify prognostic factors for regional control

Variable	**No**.	3- year RC	*p *value
Age (years)			0.146
≤ 60	32	84.4	
> 60	18	61.9	
Gender			0.093
Male	41	72.0	
Female	9	100.0	
Primary site			0.22
Nasopharynx	25	83.6	
Non-Nasopharynx	25	72.0	
T classification			0.121
1	14	68.8	
2	19	84.2	
3	8	100	
4	9	55.6	
T classification			0.05
1-3	41	82.0	
4	9	55.6	
N classification			0.43
1	12	91.7	
2	30	71.9	
3	8	75.0	
N classification			0.194
1	12	91.7	
2-3	38	72.7	
Initial maximal lymph node size (cm)			0.022
≤ 2	28	88.0	
> 2	22	63.6	
RT modality			0.887
Conventional	4	75	
3D-CRT	25	73.8	
IMRT	21	81.0	
Chemotherapy sequence			0.123
Neoadjuvant + concurrent^1^	21	71.4	
Concurrent + adjuvant^2^	17	94.1	
Concurrent only	12	64.3	
Primary tumor response			< 0.001
Complete response	38	88.1	
Partial response	10	50.0	
Progressive disease	2	0.0	
Primary tumor response			< 0.001
Complete response	38	88.1	
Non-complete response	12	41.7	
Residual maximal lymph node size (cm)			< 0.001
≤ 1.5	45	83.5	
> 1.5	5	20.0	
Lymph node necrosis			< 0.001
No	41	87.8	
Yes	9	33.3	
Follow-up PET report			0.349
Residual disease	4	50	
Normal/Reactive change	27	78.1	
Not available	19	78.9	

RC: regional control

1. Two patients who received neoadjuvant chemotherapy only were included

2. Four patients who received additional one cycle of neoadjuvant chemotherapy were included

PET as a first evaluation was performed in 31 patients. Only four of the 31 patients showed positive PET results with mSUV of 1.3, 2.8, 3.3, and 5.0, respectively. Two of them (mSUV 3.3 & 5.0) experienced regional failure. One patient had 1.7 cm necrotic lymph node with abnormal FDG uptake (mSUV 3.3) at 3 weeks post-radiotherapy. We decided that post-radiotherapy 3 weeks was too early to determine overall response and re-checked CT 8 weeks later. At 11 weeks, lymph node further decreased but was progressed in next follow-up CT. Another patient with regional recurrence had 1-cm lymph nodes without necrosis at 8 weeks after radiotherapy, and PET showed small but hypermetabolic lymph nodes (mSUV 4.2 and 5.0) at that time. Because there were small lymph nodes without necrosis, and FDG uptake decreased compared with pre-treatment value (mSUV 9.0), we observed without immediate neck dissection. However after 3 months, lymph nodes increased with necrosis and FDG uptake also increased up to mSUV 12.5, and the patients underwent salvage neck dissection. Since PET was performed in only a subset of the patients (n = 31) at the first follow-up, we analyzed subset analysis in patients who checked PET. Positive and negative PET results were reported in 4 and 27 patients, respectively, and the corresponding 3-year RC rates were 50% and 78.1% (p = 0.115). When we included patients who did not perform PET and divided patients into 3 groups (residual disease, normal/reactive change, and no PET), and 3-year RC rates were not significantly different (p = 0.349). We could not find any prognostic significance for regional control by the statistical analysis using several arbitrary cut off points of mSUVs regardless of the interpretation by the specialists in Nuclear Medicine.

## Discussion

We report the clinical outcomes of node-positive head and neck cancer patients who were treated with definitive radiochemotherapy without planned neck dissection. There are some studies reporting the results of omitting planned neck dissection in patients who obtain complete response. Their regional failure rate was generally below 10%, similar to regional control rates in planned neck dissection series [5]. Because there were no definite criteria of complete nodal response, we included all patients treated with definitive radiochemotherapy regardless of nodal response. Overall, 11 patients (22%) experienced neck failure. However, seven of these were successfully salvaged with neck dissection, and the ultimate neck control rate was 92%. Moreover, we tried to treat all recurrences aggressively, and eight of 17 patients with recurrence were alive without disease at the last follow-up. Therefore, aggressive salvage treatment should be considered if the general condition of the patient allows it.

Several studies support the policy of omitting planned neck dissections in patients who obtain a complete response to radio(chemo)therapy [10-14]. However, these studies used various definitions of "complete response" of the neck. Some studies evaluated response by physical examination, and a complete response was defined ambiguously as "not clinically detectable" [10,15]. Many studies defined complete response as complete disappearance of any detectable disease. The problem here is defining the criteria for "detectable disease". According to the definition of response evaluation criteria in solid tumors (RECIST), complete response of a lymph node is the reduction of the short axis to < 10 mm [9]. However, some studies evaluating neck response after definitive radiochemotherapy used criteria of < 1 cm or ≤1.5 cm for maximum diameter without any focal abnormality [11,13]. Because of these different criteria, we included all patients treated with definitive radiochemotherapy regardless of nodal response and analyzed factors related to regional recurrence.

As a result, initial and post-radiotherapy nodal size, post-radiotherapy nodal necrosis, and post-radiotherapy primary tumor response were associated with regional control. Post-radiotherapy nodal size of ≤1.5 cm and no necrosis have been reported as predictors of negative neck dissection pathology in a previous study [13]. In our study, there was only one regional recurrence among patients with complete response of the primary site, no evidence of residual lesion > 1.5 cm or necrotic lymph nodes at the first follow-up evaluation. The patient had 1-cm lymph nodes without necrosis at 8 weeks after radiotherapy, and PET showed small but hypermetabolic lymph nodes (mSUV 4.2 and 5.0) at that time. Because there were small lymph nodes without necrosis, and FDG uptake decreased compared with pre-treatment value (mSUV 9.0), we observed without immediate neck dissection. However, lymph nodes increased with necrosis and FDG uptake also increased up to mSUV 12.5 after 3 months and the patients underwent salvage neck dissection.

Some investigators used PET or PET/CT to detect residual neck disease and reported high negative predictive values [16,17]. In our study, PET was performed in only a subset of the patients (n = 31) at the first follow-up. Positive and negative PET results were reported in 4 and 27 patients, respectively, and the corresponding 3-year RC rates were 50% and 78.1% (p = 0.115). Since the sample size was too small to determine definite conclusion, the role of PET should be further investigated in the future.

Limitations of this study include the relatively short follow-up period, small number of patients, heterogeneous patient population and treatment regimen, and retrospective nature. Nasopharyngeal carcinoma is highly radiosensitive and mainstay of treatment is radiotherapy with chemotherapy. Despite their radiosensitivity, the incidence of persistent or recurrent neck disease has been reported to be 5-18% [18,19]. However, neck control (observation or immediate neck dissection) should be the major concern whenever response of lymph node is less than complete response after radio-chemotherapy whether primary is nasopharyngeal or non-nasopharyngeal lesion. Taking into account the different sensitivity to radiochemotherapy between nasopharyngeal and non-nasopharyngeal cancer, we analyzed primary site as one of the candidate prognostic factor. The 3-year RC rate of nasopharyngeal carcinoma and non-nasopharyngeal carcinoma was 83.6% and 72.0% respectively and difference was statistically not significant (p = 0.22). A large diversity of treatment regimens is another limitation. Chemotherapy sequence and radiotherapy modality did not show significant prognostic impact on regional recurrence.

## Conclusions

In conclusion, the neck failure rate after definitive radiochemotherapy without planned neck dissection was 22%. However, two-thirds of these were successfully salvaged by neck dissection and the ultimate neck control rate was 92%. Based on analysis of prognostic factors, patients with complete response at the primary site, no evidence of residual lesion > 1.5 cm, or no necrotic lymph nodes at the 1-2 month follow-up evaluation after radiotherapy might be spared from planned neck dissection. However, prospective evaluation in a larger group of patients would be clearly necessary before this recommendation can be included in routine management of patients.

## Competing interests

The authors declare that they have no competing interests.

## Authors' contributions

NYJ participated in the literature search, data acquisition and analysis, statistical analysis, and manuscript preparation. IAK designed the study, edited manuscript, and gave final approval for publication. IAK, KWL, SHA, and JSK provided study materials or patients. All authors have read and approved the final manuscript.

## Pre-publication history

The pre-publication history for this paper can be accessed here:

http://www.biomedcentral.com/1471-2407/12/59/prepub
